# Investigating Alternative Container Formats for Lyophilization of Biological Materials Using Diphtheria Antitoxin Monoclonal Antibody as a Model Molecule

**DOI:** 10.3390/pharmaceutics13111948

**Published:** 2021-11-17

**Authors:** Kiran P. Malik, Chinwe Duru, Paul Stickings, Esther Veronika Wenzel, Michael Hust, Paul Matejtschuk

**Affiliations:** 1 Standardization Science Section, National Institute of Biological Standards and Control, MHRA, Blanche Lane, South Mimms, Potters Bar EN6 3QG, Hertfordshire, UK; chinwe.duru@nibsc.org (C.D.); paul.matejtschuk@nibsc.org (P.M.); 2 Bacteriology Division, National Institute of Biological Standards and Control, MHRA, Blanche Lane, South Mimms, Potters Bar EN6 3QG, Hertfordshire, UK; paul.stickings@nibsc.org; 3 Department of Biotechnology, Institute for Biochemistry, Biotechnology and Bioinformatics, Technische Universität Braunschweig, Spielmannstr. 7, 38106 Braunschweig, Germany; esther.wenzel@tu-braunschweig.de (E.V.W.); m.hust@tu-bs.de (M.H.); 4 Abcalis GmbH, Inhoffenstr. 7, 38124 Braunschweig, Germany

**Keywords:** lyophilization, biologicals, formulation, stability, recombinant antibody

## Abstract

When preparing biological reference materials, the stability of the lyophilized product is critical for long-term storage, particularly in order to meet WHO International Standards, which are not assigned expiry dates but are expected to be in use for several decades. Glass ampoules are typically used by the National Institute for Biological Standards and Control (NIBSC) for the lyophilization of biological materials. More recently, a clear need has arisen for the filling of smaller volumes, for which ampoules may not be optimal. We investigated the use of plastic microtubes as an alternative container for small volume fills. In this study, a recombinant diphtheria antitoxin monoclonal antibody (DATMAB) was used as a model molecule to investigate the suitability of plastic microtubes for filling small volumes. The stability and quality of the dried material was assessed after an accelerated degradation study using a toxin neutralization test and size exclusion HPLC. While microtubes have shown some promise in the past for use in the lyophilization of some biological materials, issues with stability may arise when more labile materials are freeze-dried. We demonstrate here that the microtube format is unsuitable for ensuring the stability of this monoclonal antibody.

## 1. Introduction

Lyophilization is a widely used processing step for a range of biological materials as it allows increased long-term physical and bioactivity stability and ease in shipping in comparison to formulations in the liquid state, which can be unstable [1]. Classically, the format for such drying of final format products has been stoppered vials; however, changing demand and delivery systems (e.g., pre-filled syringes, rapid dissolving tablets for oral delivery), as well as challenges within the diagnostics industry, have meant that a range of other formats have become popular. NIBSC is a WHO Collaborating Centre and an International Laboratory for Standardization (https://www.who.int/data accessed on 15 November 2021), producing the majority of the international reference standards. These biological standards are usually purified proteins, plasma/sera, monoclonal antibodies, viruses and nucleic acids, which, because of their labile nature and the need to ensure the long-term stability of primary standards, are usually freeze-dried [2].

Container format has been shown to be an important factor in long-term stability post-lyophilization. Glass ampoules have been shown to be superior to screw cap vials in terms of maintaining an inert and dry atmosphere in storage, showing better stability results when compared to screw-cap vials, which can show moisture exchange from the stopper in storage [3,4]. In some cases, glass ampoules may not be suitable. One example is their use for infectious materials, where it is desirable to remove the risk of sharps being generated. The fill volume is also an important factor in container choice. In recent years, we have seen an increasing demand for smaller volume fills (for instance for genomic materials, where tests are used to detect minute amounts of material), resulting in the need for an alternative container format. Others have also identified new trends and challenges in formats [5]. For fill volumes ranging from 0.25–2 mL, NIBSC typically uses flame-sealed ampoules or screw cap vials. For diagnostics applications in particular, fill volumes ranging from 20–200 µL are commonly used, and these fill volumes are too small to form an adequate freeze-dried cake to cover the base of the ampoules or vials typically used at NIBSC. This results in a ‘polo effect’, with cakes being formed with holes in the middle. As a result, these are not robust and begin to break up on handling. This does not necessarily affect product stability but can result in an undesirable product appearance.

We previously investigated the use of polypropylene microtubes as an alternative container format for a range of formulations with lyocapcluster 96 format sealing mat. The suitability of these stoppering mats with a 96 well microplate format for screening formulations for freeze drying of biologicals has been shown [6]. Experience with microtubes in a 96 tube rack format was previously gained at NIBSC, with good results for extracted glycan preparations in terms of recovery and stability (not shown).

In this study, we lyophilized a diphtheria antitoxin monoclonal antibody [7] formulated in either a model “therapeutic” or “diagnostic” formulation. The purpose of the study was to further investigate the usefulness of the microtube container format, as well as to identify a suitable formulation, in order to maintain the biological activity of the antibody and to provide long-term stability.

## 2. Materials and Methods

### 2.1. Filling and Freeze Drying

For the transfection and production of the antibody, Expi293F(TM) cells (Thermo Fisher, Dreieich, Germany Cat. No. A14527) were used. Diphtheria antitoxin mAb was supplied in PBS. This was diluted to 1.7 mg/mL for lyophilization in a typical diagnostic formulation (1% sucrose/PBS) or dialyzed against a model therapeutic formulation (20 mM histidine, 5% sucrose, 0.01% Tween 20). The diagnostic and therapeutic formulations were filled and freeze dried at pilot scale as part of the stability investigation in the container formats. The mAb was poured into either 2.5 mL glass ampoules or plastic microtubes. The lyophilization was performed using a two-day freeze-drying cycle.

Type I glass 2.5 mL ampoules (Adelphi Tubes, Haywards Heath, Sussex, UK) were used at a fill volume of 250 μL using Hamilton MLab 510B (MicroLab Technologies Ltd., Hadleigh, Essex, UK). These were fitted with 13 mm diameter halobutyl rubber closures and lyophilized using a Virtis Genesis 25EL freeze dryer (Biopharma Process Systems, Winchester, UK) for the diagnostic formulation, or in a Telstar LyoBeta 15 (Azbil Telstar SA, Terassa, Spain) freeze dryer for the therapeutic formulation. Following lyophilization, the freeze dryers were back-filled with nitrogen gas and containers stoppered in situ. After removal, the ampoules were flame-sealed using an Adelphi manual sealer (Adelphi Tubes).

Plastic microtubes (MPW32054BC3, 1.4 mL, Micronics, obtained through NBS Scientific, London, UK) were filled at 50 µL per tube and lyophilized. Lyocapcluster 96 format sealing mats (Micronics MP53099) were placed loosely over the tubes; following lyophilization, the dryer was backfilled with nitrogen gas and the stoppering caps were pushed into the tubes using the dryer stoppering ram under partial vacuum around 600Torr.

After lyophilization, an accelerated degradation study was set up (storage temperatures −20, +20, +37 and 45 °C) for both the ampoules and microtubes. The stability was assessed by toxin neutralization assay at 1, 6 and 16 weeks and by SE HPLC at 1 and 16 week timepoints.

### 2.2. SE-HPLC

The stability of the product was assessed by size exclusion HPLC (Dionex Ultimate 3000, ThermoScientific Ltd., Hemel Hempstead, UK). Testing was performed at 1 and 16 week timepoints after storage at elevated temperatures. The method used was in accordance with the European Pharmacopoeia Monograph method (9th edition, vol 2, page 2688).

The freeze-dried ampoules were all taken out of ATD storage and reconstituted in 1 mL water to produce a concentration of 0.61 mg/mL for the diagnostic formulation and 1.37 mg/mL for the therapeutic formulation. The samples were then aliquoted into autosampler vials and each vial tested in triplicate.

The mobile phase included the use of 27.4 mM disodium hydrogen phosphate dihydrate, 12.6 mM sodium dihydrogen phosphate monohydrate, 200 mM sodium Chloride and 0.8 mM sodium azide in pure water. The mobile phase was vacuum filtered using a 0.2 µm cellulose nitrate membrane (Nalgene filter unit, Thermo Scientific). The temperature of the column was controlled (set to 25 °C) throughout the run. The autosampler temperature was controlled at 5 °C. The HPLC method used a 10 µL injection volume, a 0.50 mL/min flow rate, a wavelength of 280 nm and a run time 30 min. The HPLC column details were as follows: TSK gel G3000SWXL (Sigma Aldrich, Burlington, MA, USA), phase diol 5 µm, 7.8 × 300 mm, col #: 004D03343D, Lot 004D, part #: 0008541. The HPLC system used was a Thermo Scientific Dionex Ultimate 3000.

### 2.3. Vero Cell Toxin Neutralization Test to Determine Potency of the Monoclonal Antibody

The Vero cell toxin neutralization test used in this study was based on the assay first described by Miyamura et al. [8], with modifications to include spectrophotometric determination of assay end-points [9]. A complete culture medium was prepared using minimum essential medium (MEM) supplemented with 5% fetal bovine serum, 1× antibiotic-antimycotic solution, 2 mM l-Glutamine, 0.1% d-Glucose and 0.015 M HEPES. Pre-diluted mAb samples (100 μL/well) were added to the first column of a 96 well tissue culture plate (Falcon) and serial two-fold dilutions (50 μL) were prepared across the plate in complete medium. All the samples were titrated in duplicate in non-adjacent columns. The purified diphtheria toxin (NIBSC 02/154) was diluted to 2.5 × 10^−5^ Lf/mL, in complete medium, approximately four times the minimum cytopathic dose of toxin for Vero cells. WHO Vero cells (110301-D01) were used (107 Item 88020401 from ECACC, Porton, UK). The diluted toxin was added to all the wells (50 μL) containing mAb and the plates were incubated at +37 °C for 1 h for toxin neutralization to occur. At the end of the incubation period, 50 μL of a Vero cell suspension (in complete culture medium) containing 4 × 10^5^ cells/mL was added to all the sample wells. Control wells containing cells only (cell control) or cells in complete medium containing DTxn (toxin control) were included on every plate. The plates were incubated at +37 °C for 6 days. After 6 days, the cell viability was assessed using a thiazolyl blue tetrazolium bromide dye (MTT, Sigma-Aldrich, Burlington, MA, USA). At this point, 10 μL of MTT (5 mg/mL) was added per well and the plates were incubated at 37 °C for a further 4 h. The supernatants were then removed and the MTT-formazan product in the viable cells was extracted using 10% *w*/*v* sodium dodecyl sulfate in 50% *v*/*v* dimethylformamide, pH 4.7 (100 μL/well). The plates were returned to the incubator overnight to allow for the complete extraction and solubilization of the colored product, and the OD was read at 570 nm (Vmax, Molecular Devices, Wokingham, UK). The end-point for each mAb sample was determined as the last dilution showing neutralization of the toxin (defined as OD > 50% of the ‘cell only’ control wells).

## 3. Results and Discussion

### 3.1. Results of the Freeze Drying

**Figure 1:**
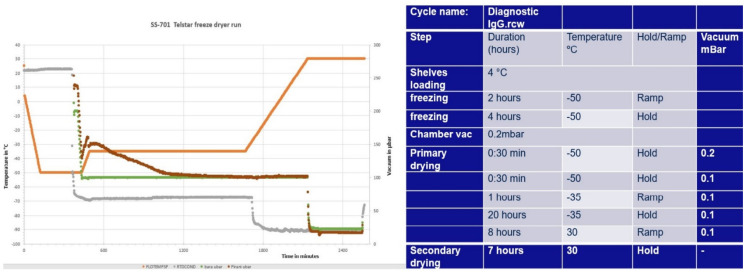
Freeze drying method and profile for SS-701 diagnostic formulation in Telstar Lyobeta freeze dryer.

For the SS-701 (diagnostic formulation), Figure 1 shows the freeze-drying cycle used. This was a two-day cycle with freeze at −50 °C, primary drying at −35 °C for 20 h, followed by a ramp over 8 h to secondary drying at 30 °C for 7 h using the pilot scale Telstar Lyobeta freeze dryer.

The run profile is also shown in Figure 1. The programmed cycle was completed as expected, suggesting that the product dried adequately over 2 days. A convergence of the vacuum pirani and baratron gauges midway through the primary drying step indicated the end of the primary drying, as the pirani gauge is sensitive to water vapor coming off during primary drying.

The therapeutic formulation was freeze dried over two days in the Virtis Genesis freeze dryer. This is a pilot scale freeze dryer similar in specification to the Telstar Lyobeta freeze dryer used for the diagnostic formulation. The freeze-drying cycle was identical, and the cycle was completed as programmed.

### 3.2. Freeze Dried Appearance Diagnostic versus Therapeutic Formulation

**Figure 2:**
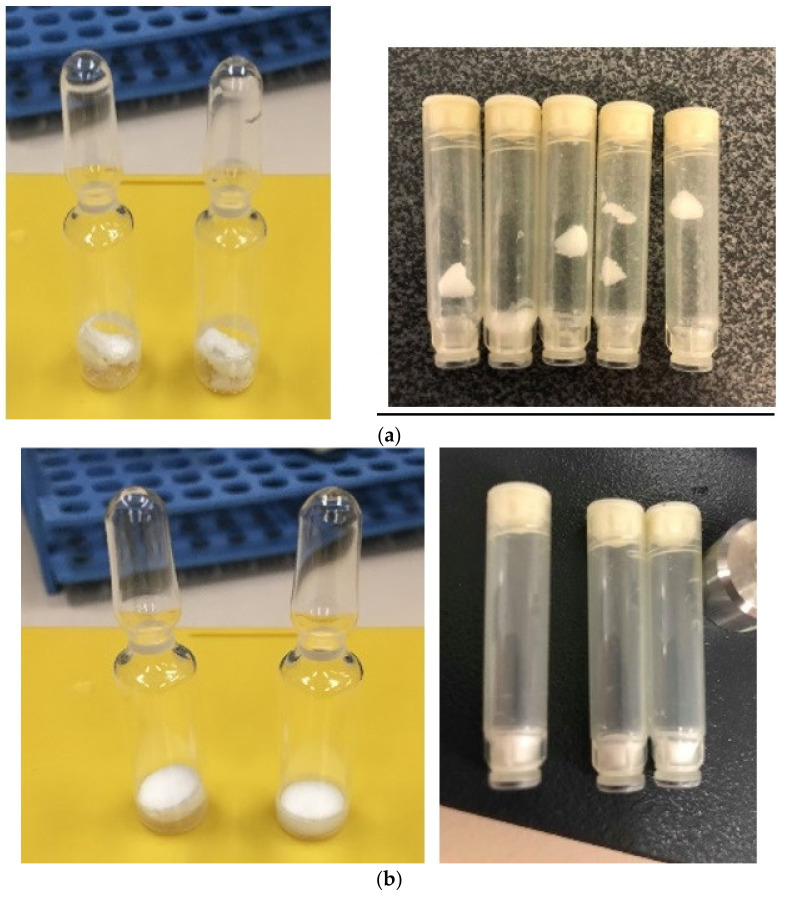
(**a**) Diagnostic Formulation freeze dried appearance in ampoules and microtubes. (**b**) Therapeutic Formulation freeze-dried appearance in ampoules and microtubes.

The appearance of both the diagnostic and the therapeutic formulations was assessed after freeze drying in terms of the robustness of the freeze-dried cake formed. Figure 2a,b show the freeze-dried products of the diagnostic and the therapeutic formulation in both ampoules and microtubes. The therapeutic formulation produced a more robust freeze-dried cake, whereas the diagnostic formulation produced a shrunken, crumbly cake, which began to break up on handling.

### 3.3. SE-HPLC Results

The SE-HPLC data are presented in Table 1 as % monomer relative to the −20°C samples at 1 and 16 week timepoints. In the microtube format, a decrease in the monomer peak was observed at elevated storage temperatures from about 94% down to 45% of the −20 °C value. At the highest storage temperature, the pre-monomer peaks increased in size and several other peaks also occurred after 20 min, suggesting the product is unstable in microtubes. On the other hand, the ampoules showed good stability after 16 weeks’ storage for diagnostic and therapeutic formulations (Table 1) when compared to the microtubes. This is demonstrated by the fact that the % monomer content was no less than 94% of the −20 °C value for all the storage temperatures for the product filled in the ampoules. However, the microtubes showed a high level of monomer loss down to about 30% of the total integrated area for both the diagnostic and the therapeutic preparations after 16 weeks when stored at 45 °C. The freeze-dried mAb was also tested after 1 week against the liquid material and no degradation was observed for any of the container formats immediately after freeze drying.

### 3.4. Results (Potency Testing)

**Figure 3:**
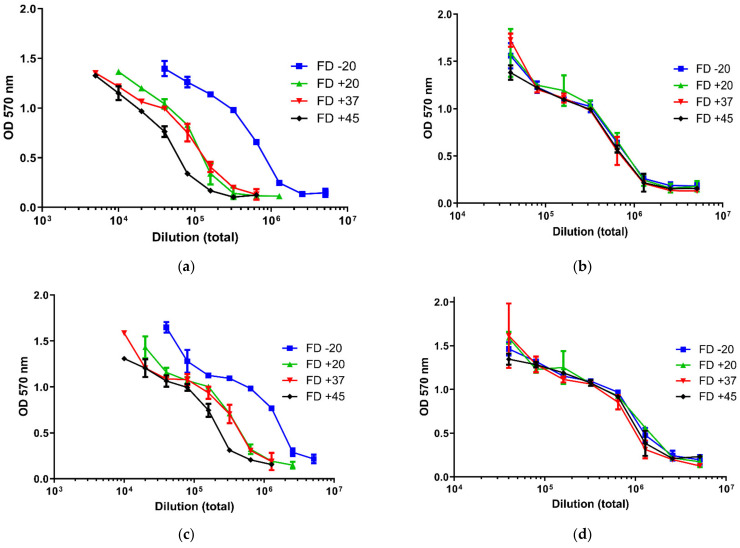
Diphtheria antitoxin potency of freeze-dried DATMAB after storage at elevated temperature at the 16 week time point. (**a**) is the diagnostic formulation freeze-dried in tubes; (**b**) is the diagnostic formulation dried in ampoules; (**c**) is the therapeutic formulation dried in tubes; (**d**) is the therapeutic formulation dried in ampoules.

The potency testing was performed using a toxin neutralization assay. No loss in potency was seen after freeze drying for either formulation or for each container type (not shown). However, after storage at elevated temperatures, there was a marked reduction in potency for both formulations for the material dried in tubes. This loss of potency for the material dried in tubes was evident at the earliest time point (1 week) used in the accelerated stability study (Table 2). The material dried in ampoules retained biological activity even after 16 weeks at elevated temperatures, whereas the material dried in tubes lost significant biological activity (Figure 3).

Both formulations of the DATMAB freeze-dried in ampoules are perfectly stable based on the biological activity and SE-HPLC profile after 16 weeks storage at elevated temperature. The results obtained for the material dried in microtubes are in stark contrast to this: the dried antitoxin in this format is clearly not stable, as shown by the Vero cell potency data and SE-HPLC profile. There was no loss of potency or change in SE-HPLC profile for the dried material compared to the pre-dried liquid for any of the formulations or container formats (not shown).

This indicates that freeze drying did not cause a loss of activity but, rather, the issue is the container format.

Further studies with more labile materials, such as Factor VIII in plasma, demonstrated that ampoules confer a much better storage stability compared with microtubes (not shown). This correlated with an increase in moisture content during storage/ATD over time with the microtubes, suggesting a compromised seal and resulting in gas exchange. As a result, microtubes are not suitable for labile biological materials prone to degradation.

## 4. Conclusions

In conclusion, the microtube format appears to be unsuitable for ensuring the stability of a freeze-dried monoclonal antibody. Based on this project and previous studies (not shown), the microtubes do not seem to be a suitable alternative for the low-volume filling of labile biological materials. However, microtubes were shown to be suitable for glycans and demonstrated an excellent stability for these more stable materials. The possible cause of the instability of the microtubes appeared to be the poor seal and/or the permeability of the plastic tube to oxygen/moisture exchange. This was also recently observed with a gDNA liquid fill, which showed satisfactory stability at sub-ambient temperatures. However, storage at 20 °C and above resulted in the evaporation and drying out of the product, indicating the seal as the likely cause.

Previous studies performed with FVIII to investigate 96 format glass tubes and also containing the plastic microtubes within sealed desiccant bags have shown no improvement with these alterations. Plastic microtubes showed an increase in moisture content on storage and a significant loss of FVIII activity. The impact of moisture content and formulation on FVIII stability has also previously been shown [10]. This also suggests that the issue lies with the integrity of the seal/closure. Glass tubes would be expected to show better moisture integrity during storage as they are not permeable; nevertheless, a loss in FVIII activity was observed. This may still have been due to leakage through the seal with the lyocapcluster. A study in which the lyocapcluster top was pushed in on a microtube with a screw cap lid that was then fitted also failed to show an improvement in long-term storage stability in plastic microtubes (data not shown). This suggests that the permeability of the microtube is critical. Investigations into the use of microtubes of different coatings or composition would also be of interest.

The production of low -volume diagnostic standards using a freeze-dried bead format [11] has been demonstrated by others, often using simple micro Eppendorf tubes as the container [12]. Here, the need to tightly control environmental conditions was in part mitigated by the use of a matrix that is insensitive to shrinkage/collapse on exposure to atmospheric moisture. It would also be of interest to try these labile biological materials in this format to compare the long-term stability.

Further studies need to focus on improving the sealing closure if microtubes are to become a viable option for low-volume fills of labile biomaterials in the future. One possibility may be their use purely for DNA standards, which are often required in very small quantities and have shown excellent stability in ampoules and vials.

For samples of larger fill volume (e.g., above 250 µL) the flame-sealed glass ampoule format was again shown to demonstrate excellent storage stability properties and remains, in our opinion, the format of choice for the long-term stable storage of labile biologics.

## Figures and Tables

**Table 1:** Diphtheria antitoxin % monomer content of freeze-dried DATMAB after storage at elevated temperature for 1 and 16 weeks. Data are the % monomer relative to −20°C samples.

“Diagnostic” Formulation—Tubes	1 Week	16 Weeks
+20 °C	96.34	77.33
+37 °C	97.84	76.46
+45 °C	89.50	44.36
**“Diagnostic” Formulation—Ampoules**	**1 Week**	**16 Weeks**
+20 °C	100.76	98.81
+37 °C	101.39	98.03
+45 °C	100.93	94.88
**“Therapeutic” Formulation—Tubes**	**1 Week**	**16 Weeks**
+20 °C	99.75	94.96
+37 °C	99.02	76.34
+45 °C	92.40	48.41
**“Therapeutic” Formulation—Ampoules**	**1 Week**	**16 Weeks**
+20 °C	100.30	102.07
+37 °C	100.17	102.16
+45 °C	99.37	102.26

**Table 2:** Diphtheria antitoxin potency of freeze-dried DATMAB after storage at elevated temperature for up to 16 weeks. Data are the potency relative to a paired sample held at the normal storage temperature of −20 °C.

“Diagnostic” Formulation—Microtubes	1 Week	6 Weeks	16 Weeks
+20 °C	1.00	0.25	0.25
+37 °C	0.50	0.25	0.18
+45 °C	0.50	0.18	0.13
**“Diagnostic” Formulation—Microtubes**	**1 Week**	**6 Weeks**	**16 weeks**
+20 °C	0.71	1.00	1.41
+37 °C	1.00	1.00	1.00
+45 °C	1.00	1.00	1.00
**“Therapeutic” Formulation—Tubes**	**1 Week**	**6 Weeks**	**16 Weeks**
+20 °C	0.35	0.50	0.18
+37 °C	0.25	0.25	0.18
+45 °C	0.18	0.25	0.09
**“Therapeutic” Formulation—Ampoules**	**1 Week**	**6 Weeks**	**16 Weeks**
+20 °C	1.00	1.00	1.00
+37 °C	1.00	1.00	1.00
+45 °C	1.00	1.00	1.00

## Data Availability

All requests for resources, reagents and data should be directed to the Lead Contact author (kiran.malik@nibsc.org).
